# Seroprevalence of severe fever with thrombocytopenia syndrome virus in southeastern China and analysis of risk factors

**DOI:** 10.1017/S0950268814001319

**Published:** 2014-05-28

**Authors:** J. M. SUN, Y.J. ZHANG, Z. Y. GONG, L. ZHANG, H. K. LV, J. F. LIN, C. L. CHAI, F. LING, S. L. LIU, S. P. GU, Z. H. ZHU, X. H. ZHENG, Y. Q. LAN, F. DING, W. Z. HUANG, J. R. XU, E. F. CHEN, J. M. JIANG

**Affiliations:** 1 Zhejiang Provincial Centre for Disease Control and Prevention, Hangzhou, China; 2 Anji Centre for Disease Control and Prevention, Anji, China; 3 Yiwu Centre for Disease Control and Prevention, Yiwu, China; 4 Xianju Centre for Disease Control and Prevention, Xianju, China; 5 Lishui Centre for Disease Control and Prevention, Lishui, China; 6 Haining Centre for Disease Control and Prevention, Haining, China; 7 Pujiang Centre for Disease Control and Prevention, Pujiang, China; 8 Xiangshan Centre for Disease Control and Prevention, Xiangshan, China

**Keywords:** Seroprevalence, severe fever with thrombocytopenia syndrome, risk factor

## Abstract

Severe fever with thrombocytopenia syndrome virus (SFTSV) has been prevalent for some time in China and it was first identified in 2010. However, the seroprevalence of SFTSV in the general population in southeastern China and risk factors associated with the infection are currently unclear. Blood samples were collected from seven counties across Zhejiang province and tested for the presence of SFTSV-specific IgG antibodies by ELISA. A total of 1380 blood samples were collected of which 5·51% were seropositive for SFTSV with seroprevalence varying significantly between sites. Seroprevalence of SFTSV in people who were family members of the patient, lived in the same village as the patient, or lived in a different village than the patient varied significantly. There was significant difference in seroprevalence between participants who bred domestic animals and participants who did not. Domestic animals are probably potential reservoir hosts and contact with domestic animals may be a transmission route of SFTSV.

## INTRODUCTION

Severe fever with thrombocytopenia syndrome (SFTS) is an emerging infectious disease discovered in China in 2010, which is caused by a novel bunyavirus, SFTS virus (SFTSV). The genome of SFTSV contains three segments of negative or ambisense polarity, designated L, M and S segments. The major clinical symptoms and laboratory abnormalities of SFTS are fever, thrombocytopenia, leukopenia, and elevated serum hepatic enzymes, and SFTS patients usually die due to multiple organ failure [1]. The clinical symptoms, however, are less specific and need to be differentiated from various infectious disease, in particular from haemorrhagic fever with renal syndrome (HFRS) caused by hantavirus and human anaplasmosis [2, 3].

In 2011–2012, 2047 cases of SFTS and 129 deaths were reported in over 206 counties of eastern and central China [4]. Cases of SFTS were also identified in Zhejiang province and a total of 65 cases were reported according to the information system for disease control and prevention in recent years [5–7]. However, to date, there have been no efforts to explore the seroprevalence of SFTSV in this region, nor to identify risk factors associated with the infection. In this study, we investigated the prevalence of SFTSV in general human populations for the first time and analysed risk factors for SFTS which will highlight the way for successful control and prevention of this emerging infectious disease.

## METHODS

### Blood samples

**Fig. 1.**
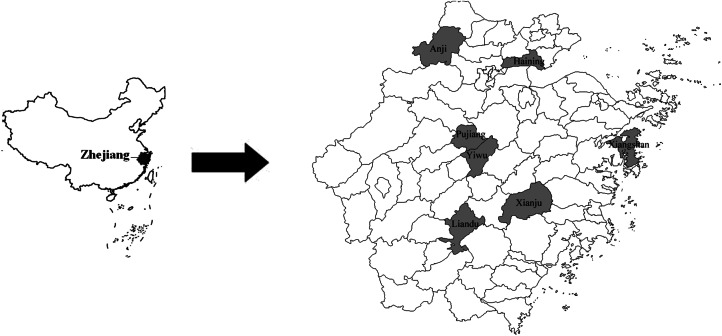
Geographical locations of the seven counties where blood samples were collected.

Zhejiang province is located in southeastern of China and is adjacent to Jiangsu and Anhui where SFTS is endemic. The investigated sites including Pujiang, Liandu, Xiangshan, Yiwu, Anji, Haining and Xianju were randomly chosen based on their geographical location and environment (Fig. 1) in Zhejiang province. Blood samples were collected from the seven locations from January to December 2013 and samples of blood serum were prepared after collection. The aims of our study were explained to all participants upon enrolment, and their consent was obtained prior to inclusion in this study. All enrolled participants provided information upon inclusion in the study with regard to their age, gender, place of residence, whether they bred domestic animals, whether they had contact with wildlife, whether they had any outdoor activities in the previous 2 weeks, and whether ticks were present in their environment.

### Serological testing

All serum samples were transported to Zhejiang CDC and stored at −80°C prior to use. Serum samples were tested for the presence of SFTSV-specific IgG antibodies using ELISA kits provided by the National Institute for Viral Disease Control and Prevention as described previously [8]. ELISA results were confirmed by immunofluorescence assay (IFA) as appropriate After the samples were diluted 1:10, 1:20, 1:40, and 1:80 in phosphate-buffered saline (PBS)-Tween buffer, the IFA was performed. Positive and negative controls were also used. Immunofluorescence was observed using an epifluorescence microscope. According to the guidelines, a titre of 1:20 was considered indicative of an infection.

### Data analysis

Logistic regression analysis, *χ*^2^ test or Fisher's exact test were used to compare SFTSV seroprevalence between sites, gender, age groups, place of residence, whether participants bred domestic animals, whether participants had contact with wildlife, whether participants had outdoor activities in the previous 2 weeks, and whether ticks existed in their environment. The difference was considered statistically significant when *P* < 0·05. Statistical analysis was performed using SPSS software v. 17.0 (SPSS Inc., USA).

The dependent variable in the logistic regression was assigned as the serological status and the independent variables were site, gender, age group, place of residence, breeding domestic animals, contact with wildlife, outdoor activities in the previous 2 weeks, and presence of ticks in their environment (Table 1). The method of logistic regression used was forward-conditional. The stepwise probability was set to 0·05 for entry and 0·10 for removal. The classification cut-off was 0·5 and the maximum number of iterations was 20. Omnibus tests of model coefficients were also conducted.

**Table 1.** Assignment of variables in logistic regression analysis

Variable	Assignment
Site	Pujiang = 1, Lishui = 2, Xiangshan = 3, Yiwu = 4, Anji = 5, Haining = 6, Xianju = 7
Gender	Male = 0, female = 1
Age group (years)	>1 = 1, >40 = 2, >55 = 3, >70 = 4
Place of residence	Family member of patient = 1, living in the same village as the patient = 2, living in a different village than the patient = 3
Breeding domestic animals	No = 0, yes = 1
Contact with wildlife	No = 0, yes = 1
Outdoor activities in previous 2 weeks	No = 0, yes = 1
Ticks present in the environment	No = 0, yes = 1
Serological status	Negative = 0, positive = 1

### Ethical statement

Experimental research reported in this study has been performed with the approval of the Ethics Committee of Zhejiang Provincial Centre for Disease Control and Prevention (Zhejiang CDC). Human research was conducted in compliance with the Helsinki Declaration.

## RESULTS

**Fig. 2.**
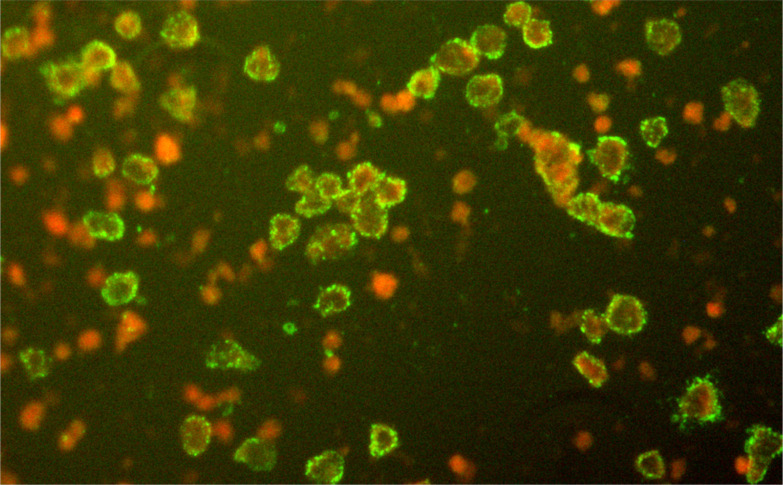
Positive results of severe fever with thrombocytopenia syndrome virus in serum samples from Zhejiang province by immunofluorescence assay.

Blood samples were collected from 1380 people living in seven locations across Zhejiang province (Table 2). Overall, 5·51% (76/1380) of blood samples were seropositive for SFTSV and seroprevalence varied significantly between sites within Zhejiang province (1·50–10·57%, *χ*^2^ = 29·607, *P* = 0·000). All 76 ELISA-positives were confirmed by IFA (Fig. 2). Seroprevalence of SFTSV was found to be similar in males and females (6·02% and 5·07%, respectively; *χ*^2^ = 0·592, *P* = 0·441 > 0·05). Furthermore, participants were divided into four age groups (>1, >40, >55, >70 years) and the seroprevalences were 7·29% (21/288), 3·10% (10/323), 3·98% (18/452), 8·52% (27/317), respectively. Of note, seroprevalence in the >70 years and >1 year age groups was significantly higher than in the >40 and >55 years age groups.

**Table 2.** Seroprevalence of SFTSV in blood samples from Zhejiang province, China

Site	Total (*n*)	Seropositive (*n*)	Prevalence (%)	*χ*^2^	*P*
Pujiang	54	4	7·41	29·607	0·000
Liandu	220	18	8·18		
Xiangshan	265	28	10·57		
Yiwu	200	7	3·50		
Anji	241	5	2·07		
Haining	200	11	5·50		
Xianju	200	3	1·50		
Total	1380	76	5·51		

SFTSV, Severe fever with thrombocytopenia syndrome virus.

Seroprevalence of SFTSV in people who were family members of the patient, lived in the same village as the patient, or lived in a different village than the patient was 18·18%, 8·96% and 4·20%, respectively (*χ*^2^ = 14·662, *P* = 0·001<0·05). Seroprevalence of people who were family members of the patient, or lived in the same village as the patient were significantly higher than that of people who lived in a different village. Moreover, there was significant difference of seroprevalence between participants who bred domestic animals and participants who did not (7·91% and 4·68%, respectively; *χ*^2^ = 5·281, *P* = 0·022<0·05). However, contact with wildlife (0% and 5·63%), outdoor activities in the previous 2 weeks (7·43% and 4·98%) and ticks in the environment (6·69% and 5·24%) were all insignificant factors in SFTSV antibody expression based on *χ*^2^ test or Fisher's exact test (Table 3).

**Table 3.** Risk factors for seroprevalence of severe fever with thrombocytopenia syndrome virus in Zhejiang province, China

Characteristics	Seronegative (*n* = 1304)	Seropositive (*n* = 76)	*χ*^2^	*P*
Male	593	38	0·592	0·441
Female	711	38		
Age group (years)				
>1	267	21	12·91	0·005
>40	313	10		
>55	434	18		
>70	290	27		
Family member of patient	9	2	14·662	0·001
Live in the same village as the patient	315	31		
Live in a different village than the patient	980	43		
Breeding domestic animals				
Yes	326	28	5·281	0·022
No	978	48		
Contact with wildlife				
Yes	31	0		0·411*
No	1273	76		
Outdoor activities in previous 2 weeks				
Yes	274	22	2·684	0·101
No	1030	54		
Ticks present in the environment				
Yes	237	17	0·841	0·359
No	1067	59		

SFTSV, Severe fever with thrombocytopenia syndrome virus.

* Fisher's exact test.

According to results of logistic analysis, the *χ*^2^ value in omnibus tests of model coefficients was determined as 20·507 (*P* < 0·05). Furthermore, the overall correct percentage was found to be 94·5%. Variables in the equation below included site and place of residence and the Wald values were determined to be 7·742 (*P* = 0·005) and 4·037 (*P* = 0·045). The equation was:


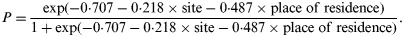


## DISCUSSION

In our study, the seroprevalence of SFTSV was found to be 5·51% in the general population, a percentage similar to the recently reported percentage (6·37%) recorded in Hubei province in China [9], but much higher than the percentage reported in Shandong province (0·84%) [10], and Jiangsu (0·94) [11]. This discrepancy may be attributed to the season of sample collection, age and sex of the subjects, previous exposure or low level of infection, and/or detection method utilized in individual studies. SFTSV antibodies were also found in Liandu and Haining where no cases were confirmed. These results suggest that subclinical SFTSV infections or a relatively mild form of SFTS illness may occur in humans. Although the seroprevalence of SFTSV varied between sites within the province, the fact that populations with SFTSV antibodies were detected across all seven study sites of Zhejiang province indicates that the general population in this region is at risk of exposure to SFTSV. Furthermore, seroprevalence in the >70 years and >1 year age groups was significantly higher. This may suggest poor immunity, but it is also possible that anti-SFTSV antibodies are long-lived, leading to accumulated antibodies in the elderly and that higher ratios in infants are due to maternal antibodies. Anti-hantavirus antibodies can last a lifetime in individuals who have been infected with hantavirus. Further studies, e.g. to study the duration of anti-SFTSV antibodies and correlation of antibody status between infants and their mothers, should be conducted to explore the reasons.

A previously published study reported that SFTSV RNA was detected in acute serum samples which were collected in 2006 indicating that SFTSV has been prevalent for some time in China [12]. However, it was first discovered in 2010 in China and the transmission cycle of SFTSV is not well understood currently. SFTSV is believed to be transmitted by ticks because the virus has been detected in *Haemaphysalis longicornis* ticks [1]. However, the disease can also be transmitted from person to person through contact with an infected patient's blood or mucous [12–15]. Here, we found that SFTSV seroprevalence in people who were family members of the patient was the highest and seroprevalence of populations who lived in the same village as the patient was significantly higher than that of populations from a different village than the patient. The reasons may be that populations in the first two groups have more chance of exposure to risk factors for SFTS. However, we should not exclude person-to-person transmission between patient and family members.

Interestingly, breeding domestic animals including dogs, cattle, goats, and chickens was a significant determinant of seroprevalence in our study. The data indicate that these domestic animals may be potential reservoir hosts of SFTSV which is consistent with the results of other studies. Niu *et al.* reported that SFTSV-specific antibodies were detected in sheep (328/472, 69·5%), cattle (509/842, 60·5%), dogs (136/359, 37·9%), pigs (26/839, 3·1%), and chickens (250/527, 47·4%) [16]. Another study in Shandong province showed 111/134 (83%) goats were seropositive for SFTSV [10], and a serosurvey of domesticated animals conducted in Jiangsu province found SFTSV antibody-positive rates of 57% in goats, 32% in cattle, 6% in dogs, 5% in pigs, and 1% in chickens but no antibodies in geese and mice [11]. Additionally, the data also suggest that populations might be infected with SFTSV via contact with secretions although this may not be the major transmission route.

Contact with wildlife and outdoor activities in the previous 2 weeks were insignificant factors for seroprevalence according to *χ*^2^ test. The reasons may be that few people have the opportunity for contact with wildlife or that wildlife is probably not a reservoir of SFTSV. Outdoor activities are not risk factors, suggesting that populations can be infected with SFTSV at home and domestic animals are probably reservoirs of SFTSV. The fact that ticks in the environment is also an insignificant factor for seroprevalence is disappointing. This result may be related to bias in the investigation as ticks are very small and many people do not recognize them. However, these data also inform us that other transmission routes may exist besides tick bites.

In summary, our study confirmed that SFTSV antibodies are widespread across Zhejiang province although patients were not identified in many regions. Populations who are family members of the patient, live in the same village as the patient, or breed domestic animals are more likely to have SFTSV antibodies than others. Furthermore, our data also inform that domestic animals are probably potential reservoir hosts and contact with patients or domestic animals may be transmission routes of SFTSV. More studies are needed to elucidate the SFTSV transmission model in nature and risk factors for human infection.
